# Medical and Physical Intelligence

**Published:** 1804-05-01

**Authors:** 


### MEDICAL AND PHYSICAL

### INTELLIGENCE.

Mr. Vauquelin has communicated in a letter to Mr. Klaproth of Berlin, the discovery of a new metallic substance, which was found mixed with the platina sand. Being engaged in experiments on platina, M. Vauquelin, Fourcroy, and Descotels traced the new metal in the residuum, which remains after dissolving raw platina in nitro-muriatic acid, (aqua regia); it was found united with chromium and iron. The above mentioned residuum, which is equally insoluble in simple as well as compound acids, was treated with caustic kali, which, after ignition, was charged with acid of chromium, and the substance that remained insoluble had a dark green colour. On treating it with concentrated muriatic acid, it was for the most part dissolved; the solution appeared of a dark green colour. By thus continuing to treat the black residuum of platina alternately with kali and muriatic acid, they succeeded in entirely decomposing it, and separating it into chromium, iron, and the new metal. As far as it has hitherto been examined, it seems to be distinguished by the following properties.

1. Its solution in muriatic acid is generally green but sometimes blue, which depends on the proportion of the oxygen.

2. This blue or green solution, when heated to the seething point, is changed into a beautiful saturated red brown colour.

3. This colour disappears instantly by the addition of sulphat of iron, and the solution again becomes green.

4. Alkalis separate the oxyd from the green solution with a green colour, and from the brown solution with a brown colour.

5. The

5. The oxyd is easily reduced without the addition of a combustible body, and the metal is white, almost like platina, and malleable.

6. From this metal, when oxydated to a certain degree which is not yet ascertained, the solution of platina acquires the property of being precipitated by sal ammonia with a red colour, because on adding to a solution of pure platina which is precipitated by sal ammonia pale green, a certain portion of the solution of the new metal, it will be precipitated with a beautiful red colour. This is likewise proved in the following manner: On pouring to a solution of the red triple salt of platina an equal quantity of caustic kali, a flaky green precipitate is formed, which is not the case in the pale yellow solution of that metal. It seems as if the common proceedings employed for the purification of platina, are insufficient to separate the new metal, as it was still found in considerable quantity in the platina metal purified by Mess. Jeanetty and Neker de Saussure, at Paris. Besides the iron, the chromium, and the new metal, the platina sand contains also the Titan metal.

Dr. KEUTSCH has found that friction with oil is highly useful in curing fevers which are peculiar to the West India islands. It produces strong perspiration, and checks the vomiting. In some cases the effect of friction is rendered more efficacious by adding camphor to the oil.

Mr. CARPUE will commence his Lectures on Anatomy and Surgery on Monday the 21st of May. Particulars may be known by applying to Mr. C. Leicester Square.

Dr. KIRBY has in the press, and will shortly publish at Edinburgh, a pocket volume containing Tables of the Materia Medica. The object of these Tables is to present a comprehensive view of the most essential circumstances respecting each article received by the three British Colleges. These are classed according to their effects, and of each are given the systematic and officinal names, native place, part used, usual form of exhibition, doses, officinal preparations, and the cases to which it is applicable. To each class is annexed a select number of formulæ, written in the new nomenclature adapted by the Edinburgh College in the late edition of their Pharmacopeia, and to the whole is subjoined a tabular view of the earthy, alkaline, and metallic salts employed in medicine, exhibiting their solubility in water, their composition, and the substances which decompose them by single and double affinity.

